# Tumor Development and Angiogenesis in Adult Brain Tumor: Glioblastoma

**DOI:** 10.1007/s12035-020-01892-8

**Published:** 2020-03-09

**Authors:** Bhavesh K. Ahir, Herbert H. Engelhard, Sajani S. Lakka

**Affiliations:** 1 Section of Hematology and Oncology, University of Illinois College of Medicine at Chicago, Chicago, IL 60612 USA; 2 Department of Neurosurgery, University of Illinois College of Medicine at Chicago, Chicago, IL 60612 USA

**Keywords:** Angiogenesis, Anti-angiogenesis therapy, Glioblastoma (GBM), Clinical trials in glioblastoma (GBM), Tumor development

## Abstract

Angiogenesis is the growth of new capillaries from the preexisting blood vessels. Glioblastoma (GBM) tumors are highly vascularized tumors, and glioma growth depends on the formation of new blood vessels. Angiogenesis is a complex process involving proliferation, migration, and differentiation of vascular endothelial cells (ECs) under the stimulation of specific signals. It is controlled by the balance between its promoting and inhibiting factors. Various angiogenic factors and genes have been identified that stimulate glioma angiogenesis. Therefore, attention has been directed to anti-angiogenesis therapy in which glioma proliferation is inhibited by inhibiting the formation of new tumor vessels using angiogenesis inhibitory factors and drugs. Here, in this review, we highlight and summarize the various molecular mediators that regulate GBM angiogenesis with focus on recent clinical research on the potential of exploiting angiogenic pathways as a strategy in the treatment of GBM patients.

## Introduction

Gliomas arising from the glial cells in the central nervous system of adult brain are the most common primary intracranial tumors and account for 70–80% of all brain tumors [1–3]. Based on the recent classification of central nervous system tumors, diffuse gliomas are categorized into four grades (I–IV) according to World Health Organization (WHO): diffuse astrocytoma (IDH mutant, WHO grade II), oligodendroglioma (IDH mutant, WHO grade II), oligoastrocytoma (IDH mutant, WHO grade II), anaplastic astrocytoma, anaplastic oligodendroglioma (IDH mutant, WHO grade II), oligoastrocytoma (IDH mutant, WHO grade III), and glioblastoma multiforme (GBM or IDH mutant WHO grade IV) [4–6]. Furthermore, among all glioma cases diagnosed, astrocytoma grade III and GBM is considered to be the most aggressive and highly invasive as they spread into other parts of the brain quickly [7]. In spite of the aggressive treatments that include surgery combined with radiation, chemotherapy [8], and biological therapy [9], glioblastoma tumors remain as an enormous therapeutic challenge with survival rates following diagnosis of 12 to 15 months with less than 3 to 5% of people surviving longer than 5 years [10]. GBM tumors are also highly vascular brain tumors with very poor prognosis [11, 12]. Several angiogenic receptors and factors are upregulated in GBM and stimulate angiogenesis signaling pathways through activating oncogenes and/or downregulating tumor suppressor genes [13]. In this review, we will review the basic mechanisms of various molecular signaling events that regulate GBM angiogenesis and explore the potential of targeting angiogenic signaling as a therapeutic strategy for brain tumor pathogenesis.

## Angiogenesis in Normal Physiology and in Tumor Progression

**Fig. 1:**
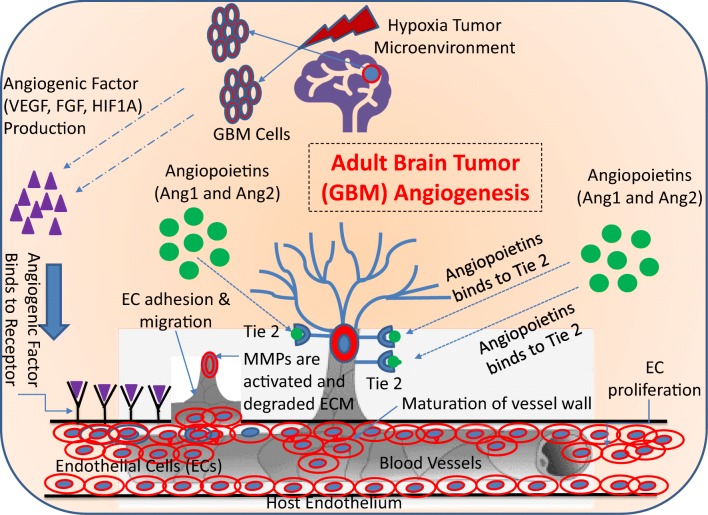
Schematic representation of angiogenic events in GBM. (a) Angiogenesis processes are initiated by the angiogenic factors, which are being released from the GBM cells in the hypoxic tumor microenvironment. The major angiogenic factors are involved in GBM angiogenesis process which includes the VEGF, FGF, HIF1α, and Ang-1 and Ang-2. (b) These angiogenic factors bind to their receptors on endothelial cells and then start to initiate the endothelial cell proliferation and migration. During the endothelial cell proliferation and migration processes, the ECM start to degrade, and the endothelial cells are assembled into a tube/vessel-like structure. (c) The final step of GBM angiogenesis process is the maturation of the blood vessel wall, which is constructed by the recruitment of pericytes to cover the endothelial cells from its outside to form a new blood vessel formation

Physiological angiogenesis is a highly regulated process and is an essential one for the adequate supply of nutrients and oxygen to developing or healing tissues [14]. It is composed of many steps and is a combination of various components such as cells (endothelial cells and mural cells), soluble growth factors, proteolytic enzymes and adhesion proteins and matrix components (ECM) as shown in Fig. 1 [15, 16]. Hypoxia (low oxygen tension) is the main trigger which induce the activation of transcription factor, hypoxia-inducible factor-1 (HIF-1), which controls the expression of growth factors [17], matrix components [18, 19], adhesion molecules [20], and metabolic proteins [21]. The induction of angiogenesis relies on a balance between pro- and anti-angiogenic factors. Lack of oxygen in the cell simulates the release of pro-angiogenic growth factors like vascular endothelial growth factor (VEGF) [22], transforming growth factor-β (TGF-β) [23], fibroblast growth factors (FGFs) [24], angiopoietin-1 [25], and epidermal growth factor (EGF) [26]. These angiogenic factors bind to their receptors on the endothelial cell membrane resulting in the dissolution of the vessel wall and degradation of the endothelial cell basement membrane and extracellular matrix (ECM). Following the degradation of the basement membrane, specific proteases such as matrix metalloproteinases (MMPs) remodel the extracellular matrix components and a new matrix is synthesized by stromal cells which in turn foster the migration and proliferation of endothelial cells resulting in the formation of an endothelial tube-like structure [27]. Finally, a mature vascular basement membrane is formed around this newly formed the endothelial tube and the Mural cells (pericytes and smooth muscle cells) surrounding it resulting in a stable new vessel (Fig. 1) [28, 29].

Angiogenesis is essential for tumor growth and progression. The tumor cells away from vessels experience hypoxia due to deficiency of blood and oxygen. Hypoxic environment induces cancer stem cells (CSCs) to differentiate toward endothelial progenitor cells and mature endothelium, which in turn generates new blood vessels inside the tumor. Tumors generate abnormal and functionally immature blood vessels due to deregulated factors such angiogenic growth factors, angiogenesis inhibitors, and other genetic factors by a process known as pathological angiogenesis [30]. Blood vessels developing in the primary tumor are larger than their normal counterparts and follow a criss-cross path, with irregular lumen diameters, dilated, highly permeable, and branch irregularly [31]. The tumor vasculature is also hyperpermeable to plasma and plasma proteins leading to local edema and extravascular clotting of plasma [32, 33]. This increase in the interstitial pressure in the tumor vasculature alters the blood flow and flux of leukocytes reaching the tumor site [34]. In addition, tumor cells can easily spread to the distant tissues due to the defective basement membrane and lack of normal perivascular connective tissue barrier [35]. Leakiness and compression of vessels leaves large volumes of tissue without blood flow in tumor and obstructs the delivery of blood-borne drugs, oxygen, and nutrients resulting in ischemia and necrotic regions within the tumor [36–38]. Ischemia leads to a hypoxic environment which in turn activates the HIF-1 resulting in new blood vessel formation [39]. Thus, the disorderly grown tumor vasculature observed in the tumors dramatically alters the tumor microenvironment and influences various aspects of tumor progression like tumor growth, allows easy penetration of the tumor cells and its ability to metastasize to distant sites, escape from the host immune system and response to anticancer therapies. Given the role of angiogenesis in tumor growth, targeting tumor vasculature and inhibition of growth factors/signaling pathways necessary for endothelial cell growth and proliferation is one of the practical approaches to inhibit tumor angiogenesis.

## Factors Involved in Brain Tumor Angiogenesis

**Fig. 2:**
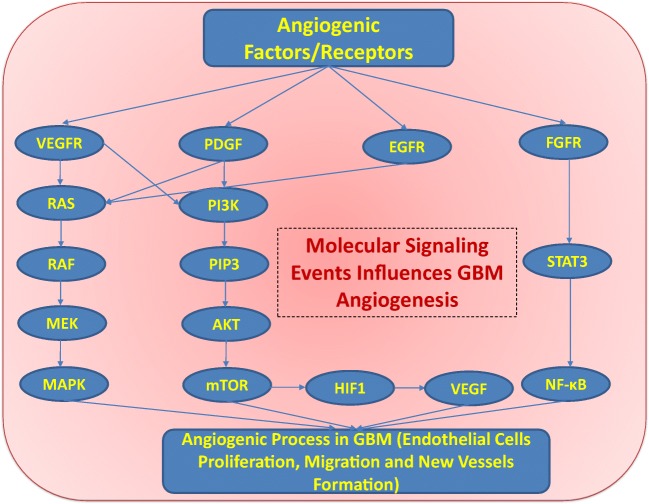
Angiogenic factors and receptors involved in GBM angiogenesis. VEGFR, PDGF, EGFR, and FGFR involved in the key molecular signaling events (RAS/RAF/MEK/MAPK signaling pathway and PI3K/AKT/mTOR signaling pathway) which plays an important role in glioma cells proliferation, migration, and survival

Brain tumor progression is closely associated with the formation of new vessels. Brain tumor angiogenesis is mediated through the action of many angiogenic factors, some of which are involved in normal angiogenesis (Fig. 2). The best-known angiogenesis regulators in GBM progression include VEGF, basic fibroblast growth factor (bFGF), hepatocyte growth factor (HGF), platelet-derived growth factor (PDGF), and TGF-β, MMPs, and angiopoietins (Angs). The expression levels of the angiogenic growth factors were shown to impact tumor progression. These angiogenic factors are upregulated by a variety of mechanisms like oncogene activation, loss of tumor suppressor gene function, and/or hypoxic microenvironments [40]. Moreover, fibroblast growth factor receptor (FGFR) modulates a series of angiogenic processes which includes FGF-mediated glioma endothelial cell migration and proliferation. In addition, FGFR plays an important role in the survival and angiogenesis of GBM cells through phosphatidylinositol 3-kinase (PI3K)/protein kinase B or AKT/mammalian target of rapamycin (mTOR) molecular signaling pathway [41–45]. FGF1, FGF2, and FGFR also activates the c-JUN/p38-MAPK pathway and STAT3/NF-κB signaling pathway; hence, all of these molecular signaling events are the most important events associated with GBM tumorigenesis, cell proliferation, migration, and angiogenesis [41–45] (Fig. 2). Previously, it has been reported that FGF2 is a prognostic biomarker of GBM patients [44]. All these different molecular effectors interact using various receptors equipped with tyrosine kinase activity on the endothelial cells membrane and transduce signals to activate multiple signaling pathways in GBM [46]. These signal transduction pathways regulate proliferation, migration, and differentiation of endothelial cells required for new vessel growth [47]. Moreover, the combination of VEGF-A with FGF-2 with/or without platelet-derived growth factor BB (PDGF-BB) [48], and that combination of FGF-2 and PDGF-BB [49] demonstrated synergistic effect in inducing neovascularization in vivo. The expression of these growth factors correlates with tumor progression with higher-grade tumors expressing higher levels of growth factors and their corresponding receptors when compared to low-grade tumors. Those factors that are well characterized in GBM neovascularization are summarized in Table 1 and are described below.

**Table 1:** List of major angiogenesis factors in GBM

Angiogenesis factors	Molecular functions	Reference
VEGF (VEGF-A, VEGF-B, VEGF-C, VEGF-D)	It promotes endothelial cell proliferation, migration, mitosis of endothelial cells and promotes blood vessel formation (angiogenesis process).	[158–160]
VEGFR (VEGFR1, VEGFR2 and VEGFR3)	Hematopoiesis process, promotes tumor angiogenesis, activates MMPs, Mediates the angiogenic, mitogenic and permeability-enhancing effects of VEGF.	[158]
MMP-2 and MMP-9	It has been predominately involved in the proteolytic degradation of ECM components and facilitates cell motility, cell invasions and promotes glioma cells angiogenesis.	[161, 162]
aFGF and bFGF	It induces the endothelial cell proliferation and promotes tubule-like morphology in endothelial cells.	[160, 163]
FGFR	It modules the cell proliferation, cell migration and angiogenesis	[44, 160, 163]
Integrin ανβ3 and Integrin ανβ5	It facilitates the cell-to-cell interaction, cell adhesion to extra cellular matrix and cellular migration	[164, 165]
Angiopoietin 2 and Angiopoietin 4	Angiopoietin 2 binds to tyrosine kinase with immunoglobulin like and EGF like 2 (TIE-2) and it destabilizes tumor vasculature. Angiopoietin 4 binds to TIE-2 and induced angiogenesis via ERK ½ pathway.	[92, 160]
HGF	It promotes angiogenesis through induction of VEGF signaling.	[158, 160]
EGFR	It stimulates VEGF production in GBM cells	[166]
TGF-β	It promotes VEGF induced angiogenesis; it regulates endothelial cell proliferation, migration, differentiation and extracellular matrix synthesis in endothelial cells.	[167]

*aFGF* acidic fibroblast growth factor, *bFGF* basic fibroblast growth factor, *FGFR* fibroblast growth factor receptor, *HGF* hepatocyte growth factor

### VEGF

Angiogenesis is fueled by several pro-angiogenic cytokines in malignant glioma, among which VEGF is the most important signaling molecule. This family of cytokines has six VEGF isoforms (VEGF-A, VEGF-B, VEGF-C, VEGF-D, VEGF-E, and placental growth factor) [22]. VEGF-A is considered the main mediator in hypoxia-induced tumor growth. VEGF signaling is mediated through the receptor tyrosine kinases like VEGFR-1, VEGFR-2, and VEGFR-3 and mediates a variety of functions including pro-angiogenic activity, vascular permeability activity, and stimulate endothelial cell migration [50]. VEGF was shown to synergize with many growth factors and the effects of VEGF combinations with other factors exceeded those exerted by each factor alone in inducing angiogenesis [51, 52]. Binding of VEGF to its receptors on the endothelial cell membrane activates endothelial cells to secrete MMP into the surrounding tissue that are responsible for breakdown of ECM required for their proliferation and migration [53]. In addition, the combination of VEGF-A with FGF-2 or PDGF-BB was shown to have a potent synergistic effect in inducing angiogenesis in vitro and in vivo [48, 49].

VEGF plays an important role in the survival and proliferation of gliomas. VEGF mRNA expression was observed in low-grade gliomas with further upregulation in high-grade gliomas [54, 55]. Glioma formation occurs with the induction of VEGFR-1 mRNA in endothelial cells while progression toward malignancy is observed with the coordinated function of both the VEGFR-1/VEGFR-2 genes [56]. High levels of VEGF mRNA expression were observed in the necrotic regions in glioblastoma tumors [57, 58] which in turn promotes vascular proliferation and tumor progression of human glioblastoma [34, 59]. Overexpression of VEGF and VEGF-R1 in the low-grade astrocytomas was significantly associated with the same dismal prognosis as high-grade lesion, suggesting that VEGF and VEGFR expression can serve as a prognostic biomarker and provide useful information in determining the regime [60].

### FGFs

FGF is another pro-angiogenic growth factor, which is both present the tumor cells and as well as stored in the vascular basement membrane for sustained release and is upregulated during angiogenesis. Two forms of FGFs, FGF-1 or acidic FGF (aFGF or FGF1) and FGF-2 or basic (bFGF or FGF2), bind most commonly to the receptor tyrosine kinases FGFR-1 or FGFR-2 [61]. FGF binding to its receptor activates signaling pathways mediated in part by protein kinase-C (PKC), phospholipase A2 [62], and increases endothelial cell migration and capillary formation promotes capillary morphogenesis [63]. FGF-2 also mediates proteolysis of matrix components and enhances the synthesis of collagen, fibronectin, and proteoglycans by endothelial cells demonstrating its effects on ECM remodeling during angiogenesis [63].

FGF-2 is implicated in brain tumor progression and localizes in the microvasculature as well as in the tumor cells in human gliomas [64–66]. bFGF levels correlate with the degree of glioma malignancy and vascularity as determined by immunohistochemical analysis [65]. It has previously shown that antibodies against bFGF were shown to inhibit glioma growth in vivo model and led to reduced blood vessel densities in glioma tumors of treated animals [67].

### PDGF

PDGF family proteins are 45-kDa molecules and consist of four polypeptide chains (PDGF-A, PDGF-B (c-Sis form), PDGF-C, and PDGF-D) and were originally purified from platelets. All the PDGF family polypeptides have a highly conserved growth factor domain, called the PDGF/VEGF homology domain involved in forming bisulphite bridges to form the PDGF dimers PDGF-AA, PDGF-AB, PDGF-BB, PDGF-CC, and PDGF-DD [68]. PDGF proteins regulate angiogenesis by binding to and activating two cell surface receptor tyrosine kinase (RTK) receptors, PDGFR-α and PDGFR-β, which leads to receptor dimerization, transphosphorylation, and subsequent activation of intracellular signaling pathways, such as PI3K/AKT and RAS/MAPK [69]. PDGF-B and PDGFR-β axis stimulates the proliferation of cultured smooth muscle cells and pericytes to the site of newly sprouting vessels and aids in establishing a new basement membrane [70]. In addition, PDGF-BB-induced erythropoietin (EPO), a hormone that stimulates erythropoiesis is elevated during tissue hypoxia through activation of the HIF-1α and promotes angiogenesis, vascular stability, and endothelial cell survival [71, 72]. Therefore, PDGF exert its pro-angiogenic effects by direct induction of endothelial cell proliferation and new vessel formation, and by endocrine stimulation of extramedullary hematopoiesis leading to increased oxygen perfusion and protection against tumor-induced hypoxia.

Several studies demonstrate that gliomas express all the PDGF ligands [73–75]. It was suggested that the growth factors produced by endothelial cells, such as PDGF-BB attract the glioma cells to the surrounding vasculature [76]. It was observed that the expression of PDGF ligand correlates with poor prognosis factors such as age at GBM diagnosis, phosphatase and tensin homolog deletion (PTEN), and isocitrate dehydrogenase 1 (IDH1) mutation in glioblastoma patients [77]. In situ hybridization studies indicated differential expression of the PDGF ligands and their receptors in glial cell of the tumor mass and the endothelial cells in the tumor areas suggesting the presence of autocrine and paracrine stimulatory loops affecting glioma angiogenesis. High expressions of both PDGF-B and PDGFR-β mRNA were found in the endothelial cells present in the tumor tissue; these were thought to stimulate the autocrine loop with the PDGFR-β receptor, while PDGF-A mRNA and PDGF-α were observed only in the glial tumor cells stimulating the autocrine/paracrine loop with the PDGFR-α receptor [73]. PDGFR-β is preferentially expressed in GBM stem cells, and genetic or pharmacological targeting of PDGFR-β (not PDGFR-α) attenuated glioma stem cell (GSC) self-renewal, survival, and GBM progression [78, 79].

### HGF/SF

Hepatocyte growth factor/scatter factor (HGF/SF) is a heparin-binding mesenchyme-derived cytokine consisting of a 60-kDa α-chain and a 30-kDa β-chain. It transduces signals by binding to its receptor and is a transmembrane tyrosine kinase encoded by c-MET. SF and c-MET are strongly increased in several tumors and is often associated with poor prognosis [80]. HGF is a potent angiogenic molecule, and its angiogenic activity stimulates endothelial cell proliferation and migration in vitro and increases organization into capillary-like tubes in vivo. HGF/SF regulates angiogenesis by simultaneous upregulation a VEGF, a pro-angiogenic factor and suppressing thrombospondin 1 (TSP-1), an endogenous inhibitor of angiogenesis [81]. HGF/SF can also induce angiogenesis independently of VEGF through the direct activation of the AKT and ERKs to induce endothelial proliferation [82].

SF/HGF and its receptor tyrosine kinase c-MET are expressed in brain tumors and were shown to promote tumor proliferation, migration, invasion, and angiogenesis. This ligand-receptor pair expression levels correlate with tumor grade, tumor blood vessel density, and poor prognosis [83]. Inhibition of SF/HGF and c-met expression anti-SF and anti-c-MET U1/ribozymes promotes tumor cell apoptosis and inhibits tumor angiogenesis in an in vivo glioma model [84]. Suppression of both MET and VEGF exhibited a synergistic effect in the inhibition GBM growth compared to single treatment alone in an intracranial glioma mode [85].

### Ang

The angiopoietins are glycosylated proteins that bind to Tie-2 (Tyr kinase with Ig and epidermal growth factor homology domains) receptors [86]. Four types of angiopoietins have been identified (Ang-1 to Ang-4) and were shown to play a role in angiogenesis. All the angiopoietins bind the same receptor, tunica interna endothelial cell kinase 2 (Tie-2), but appear to have differential and counteracting effects on the vasculature. Ang-1 induced new vessel formation with angiogenic actions that are distinct from VEGF and stabilizes them through reciprocal interactions between the endothelium and surrounding ECM [25]. Ang-2 is upregulated by the both hypoxia and VEGF and enhances the VEGF-mediated endothelial cell migration and proliferation. In the absence of VEGF, Ang-2 functions as an antagonist to Ang-1 which mediates blood vessel regression and contributes to leakiness and fragility of tumor vessels [87]. Therefore, Angs induce context dependent pro- or anti-angiogenic effects. Furthermore, it has been established that tetrameric or higher orders of aggregation of Angs is required for Tie-2-mediated signaling, suggesting the presence of monomeric or dimeric angiopoietins that may bind to their receptor and serve as inhibitors of Tie-2 [88]. Ang-3 is maintained as a monomeric form and exerts anti-angiogenic and anti-cancer activity [89]. A study reported by Cam et al. [90] showed that targeting the angiopoietin 1 (ANGPT1)/Tie-2 axis by using a highly potent, orally available small molecular inhibitor (rebastinib) in GBM extents survival. In addition, rebastinib (DCC-2036) is a selective inhibitor of the Tie-2 immunokinase and currently in clinical trials in combination with carboplatin (NCT03717415) or paclitaxel (NCT03601897) in patients including with GBM [90].

Ang-1 mRNA was localized in tumor cells while Ang-2 mRNA was detected in endothelial cells and causes blood vessel dissolution/destabilization, and it is identified as one the early marker of glioma-induced neovascularization [66, 91]. Ang-4 is upregulated in human GBM tissues and cells and was shown to have a more potent pro-angiogenic activity than Ang-1 and promotes intracranial growth in mouse model [92]. Tie-2 expression was observed in malignant human gliomas [93], and Ang-2 regulates VEGF expression at the transcriptional level in Tie-2-expressing glioma cells [94]. One preclinical study has demonstrated that combined anti-VEGF/anti-Ang-2 therapy can obliterate resistance to VEGF monotherapy by upregulation of Ang-2 in endothelial cells and had a synergistic effect in overall GBM survival [95].

### TGF-β

The TGF-β family of structurally related polypeptides and control several pro-tumorigenic functions like proliferation, apoptosis, differentiation, epithelial-mesenchymal transition (EMT), and angiogenesis. They signal through heteromeric complexes of type I (activin receptor-like kinases, also known as TβRI) and type II (TβRII) transmembrane serine/threonine kinase receptors serine/threonine kinase receptor complexes which in turn triggers phosphorylation of the intracellular effectors, Smads (derived from proteins “Sma” and “Mad” from *C. elegans* and *D. melanogaster*) to regulate the expression of TGF-β target genes [96]. TGF-β1 is the most frequently overexpressed in carcinomas and elevated TGF-β activity has been associated with poor clinical outcome [97]. Smads interact with and modulate the functions of various transcription factors which mediate tumor-induced angiogenesis [98]. TGF-β regulates the expression of various ECM components that play a pivotal role in both the initiation and resolution phase of angiogenesis [99]. TGF-β modulates the levels of FGF-2 which is required in the formation of capillaries during angiogenesis by suppressing the induction of a serine protease, urokinase plasminogen activator [100]. TGF-β acts in concert with VEGF promote endothelial cell apoptosis as part of capillary acts in concert with TGF-β1 to induce endothelial cell apoptosis [101]. TGF-β pathway also activates αvβ3, which binds to various secreted ECM proteins, such as von Willebrand factor, TSP-1, fibrinogen, proteolyzed collagen, fibronectin, and vitronectin and facilitates their degradation during vascular remodeling during angiogenesis [102]. Several approaches have been used to neutralize TGF-β signaling at distinct levels to suppress tumor growth and angiogenesis [103].

A high level of TGF-β correlates with poor prognosis in GBM and enhances the expression of several pro-angiogenic factors such as VEGF, FGF, and PDGF-β [104]. TGF-β1 increased glioma-induced angiogenesis via JNK pathway in zebrafish embryo/xenograft glioma model [105]. A cross-talk between TGF-β and VEGF/PLGF signaling in glioblastoma was shown to have both pro- and anti-angiogenic activities in human brain-derived microvascular endothelial cells (hCMECs) and glioblastoma-derived endothelial cells (GMECs). TGF-β induces VEGF and placental growth factor (PlGF) mRNA and protein expression in glioma cells inducing pro-angiogenic effects. In contrast, exogenous TGF-β had inhibitory effects on endothelial properties and induces endothelial-mesenchymal transition (EndoMT) in hCMEC and GMEC [106]. High levels of TGF-β work in conjunction with the PDGF-β to increase GSC proliferation [104]. TGF-β induces generation of pericytes from the GSC residing in the perivascular niches to support vessel formation and tumor growth [107].

### MMPs

MMP are a family of zinc-dependent endopeptidase endopeptidases that selectively degrade components of the ECM and are implicated in tumor cell invasion angiogenesis and suppression of anti-tumor immune surveillance. An integral part of the angiogenic process is degradation of the vessel basement membrane and surrounding ECM which facilitates the invasion of endothelial cells. MMPs were also shown to stimulate the proliferation and activation of pericytes through the release of growth factor bound to the ECM and aid in their migration to the new formed vessels leading to vessel stabilization [108].

Gelatinase-A (MMP-2) and gelatinase-B (MMP-9) are highly expressed in patients with WHO grade III brain tumors [109]. Both these proteases were shown to have a synergistic effect on endothelial basement membrane degradation in gliomas [110]. MMP-9-mediated liberation of matrix-sequestered VEGF induced the angiogenic switching in a pre-malignant tumor; this effect was observed in several transgenic mouse models including glioblastoma [111].

## Angiogenic Regulators and Targets for Anti-angiogenesis Therapy in GBM

It has been previously mentioned that angiogenesis is one of the most obvious hallmarks of most tumors including adult brain tumor (GBM), which significantly contrasts GBM from normal brain tissues [112, 113]. Hence, anti-angiogenesis therapy has become the most effective strategy in the treatment of GBM patients. Previously, it has been shown that VEGF plays an essential role in the angiogenesis of GBM, and inhibiting the expression of VEGF always known to be the most effective therapeutic strategy to GBM growth in patients [58, 114]. Moreover, vasculogenic mimicry (VM) is a newly discovered tube-like vascular structure which was found to be among the potential therapies for GBM [115]. Additionally, anti-angiogenesis by the VEGF mono-antibody, bevacizumab, showed minimal efficacy and enhanced tumor invasiveness triggered by hypoxia induction, which may be partially due to VM. Several studies have reported that VM is endothelial cell-independent, consisting of tumor cells and extracellular matrix, and is found to be associated with poor prognosis in GBM patients [11, 116, 117]. In addition, these studies showed that the VM-associated mechanisms offered new insights compared to classical anti-angiogenesis therapies. These studies have also confirmed that there were a series of genes including molecular targets and molecular signaling pathways were involved in VM [115]. Hence, these molecular mechanisms of VM may provide potential targets for anti-angiogenesis therapy in GBM. For example, VEGFR-2 kinase inhibitors (SU1498 and AZD2171) have been shown to reduce VM formation in GBM cell lines in vitro and in vivo, accompanied by reduction in chemotaxis, cell proliferation, and tumorigenicity [118].

It has been reported that hypoxia-inducible gene 2 (HIG2) is a marker of hypoxia and it can serve as a diagnostic biomarker for several cancers including GBM, as a potential target for anti-angiogenesis therapy [119]. Furthermore, Mao et al. [120] showed a positive correlation of HIG2 with VEGFA and HIF1α expression, which ultimately contributes to bevacizumab resistance in GBM [120]. Several studies have shown that STAT3 is a receptor that is activated by ligand interaction and overexpression of STAT3 constitutively activated in several tumors including GBM [121, 122]. Additionally, it has been shown that STAT3 inhibitor (AZD1480) combined with cediranib significantly reduced the volume and microvessel density of GBM, suggesting that the STAT3 molecular signaling pathway may mediate resistance to anti-angiogenic therapy, and regulating the STAT3 pathway might be useful in treating the condition in GBM patients [123]. Previously, it has been shown that the downregulation of HIF1α and mTOR signaling pathway through rapamycin, including mTOR siRNA, may inhibit VM formation in GBM [124]. Moreover, this study provides the evidence that mTOR as a potential therapeutic target in GBM. A study reported by Nicholas et al. [125] has shown that the epidermal growth factor receptor (EGFR) is associated with tumor growth and angiogenesis, and it is also found activated in all types of tumors including GBM [125]. In addition, this study also reveals that RAS/MAPK and PI3K/AKT/mTOR molecular signaling pathway regulates glioma cell proliferation, differentiation, tumor angiogenesis, and survival in GBM [42, 45]. Furthermore, targeting of the RTK/PI3K/AKT pathway enhances the cytotoxic effect of radiation and TMZ in malignant GBM cells [126].

A study reported by Francescone et al. [127] showed that targeting VEGFR2 using Flk-1 shRNA in GBM-derived cell lines significantly reduced VM formation and subsequently inhibited the development of tumors [127]. In addition, the results of this study suggest that the VEGFR2 plays an important role in the formation of VM in GBM as a possible therapeutic target [127]. There were several studies demonstrate that vincristine promotes an anti-angiogenic effect via the inhibition of HIF1α in GBM, and result of this study may provide a new therapeutic target for anti-angiogenesis therapy in GBM [128, 129].

A study reported that the PTEN molecular signaling act as a tumor suppressor gene, and it is often inactivated in several cancers including GBM [130]. This study also reveals that the loss of the PTEN signaling leads to VEGFR2 expression in tumor cells in GBM patients, which may contribute to resistance against anti-angiogenic treatments. Moreover, it has been shown that overexpression of VEGFR2 in tumor cells could develop early resistance to chemotherapy with TMZ and anti-angiogenesis therapy with bevacizumab, in GBM [131].

More and more emerging studies have suggested that the targeted gene knockout techniques with well-designed experimental strategy could be effective in the treatment of GBM patients and other human diseases. Moreover, newly designed targeted drug delivery systems circumvent multidrug resistance and demonstrated an enhanced efficacy for GBM patient [132, 133]. Additionally, the use of strategies targeting multiple molecular signaling pathways in a combination with drug targets may lead to increased therapeutic efficiency, and studies on VM as a novel and distinct regulating target contribute significantly to the future of anti-angiogenesis treatment in GBM patients.

## Clinical Trials of Angiogenesis Targets in GBM

Several preclinical studies suggested that anti-angiogenic therapeutic agents enhance the efficacy of conventional treatments. A number of anti-angiogenic therapies have been evaluated in clinical trials as an alternative or complementary to conventional cancer treatments. Table 2 summarizes clinical drug trials targeting angiogenesis in primary and secondary brain tumors, and the detail information about the clinical trial, drug target concentration, number of patient population, and the current clinical trial phase for the drug approval process. Most of the anti-angiogenic agents currently in phase I/II trials for brain tumors target the VEGF pathway as VEGF family and its receptors function as the central signaling pathway of glioma angiogenesis. On this basis, the majority of these clinical trials are targeting VEGF signaling (Table 3) with monoclonal antibodies against VEGF-A (bevacizumab), a small-molecule tyrosine kinase inhibitors (TKIs) that inhibit VEGFR-2 tyrosine kinase activity (cediranib, sunitinib, vandetanib) and soluble decoy receptors developed from VEGFR-1 that selectively inhibit VEGF activity (aflibercept).

**Table 2:** Representative clinical trials of anti-angiogenic drug targets in GBM

Clinical trial no.	Antiangiogenic drug targets	Clinical trial phase	Clinical trial institution	Brain tumor disease type	Number of patients enrolled (*n*)	Concentration	PFS-6 (%)	Median OS (months)	Reference
1	Bevacizumab (BEV)	II	BRAIN	Recurrent GBM	85	10 mg/kg every 2 weeks	43	9.3	[137]
	Bevacizumab + Irinotecan	II	BRAIN	Recurrent GBM	167	10 mg/kg of BEV + Irinotecan (340 mg/m^2^ or 125 mg/m^2^) every 2 weeks	50.3	8.7	[137]
2	Bevacizumab (BEV)	II	NCI	Recurrent GBM	48	10 mg/kg every 2 weeks	29	7.8	[134]
3	Bevacizumab (BEV) + Lomustine	II	BELOB	Recurrent GBM	153	10 mg/kg every 2 weeks + 110 mg/m^2^ once every 6 weeks	42	12	[138]
4	Bevacizumab (BEV) + Lomustine	III	EORTC 26101	Recurrent GBM	437	10 mg/kg every 2 weeks +110 mg/m^2^ once every 6 weeks	Not recorded	9.1	[139]
5	Cediranib	III	REGAL	Recurrent GBM	118	30 mg daily given one time	16	8	[168]
		III	REGAL	Recurrent GBM	325	30 mg daily + 110 mg/m^2^ once every 6 weeks	35	9.4	[168]
		III	REGAL	Recurrent GBM	325	30 mg daily + Cediranib matched placebo	25	9.8	[168]
6	Enzastaurin	III	Phase-III Enzastaurin Study	Recurrent GBM	266	500 mg daily	11.1	6.6	[169]
			Phase-III Enzastaurin Study	Recurrent GBM	266	500 mg daily + 110 to 130 mg/m^2^ once every 6 weeks	19	7.1	[169]
7	Aflibercept	II	Phase-II Aflibercept Study	Recurrent GBM	42	4 mg/kg every 2 weeks	7.7	9.8	[170]
8	Nintedanib	II	Phase-II Nintedanib Study	Recurrent GBM	13	200 mg twice daily	4	8.1	[171]
9	Pazopanib	II	Phase-II Pazopanib Study	Recurrent GBM	35	800 mg daily	3	8.8	[172]
10	Pazopanib	I/II	Phase I/II Pazopanib plus Lapatinib Study	Recurrent GBM	41	400 mg daily plus 1000 mg/day Lapatinib	7.5	Not recorded	[173]
11	Sorafenib	II	Phase II study of Sorafenib Study	Recurrent GBM	32	400 mg daily	9.4	10.4	[174]
		II	Phase II study of Sorafenib Study	Recurrent GBM	32	400 mg daily plus TMZ daily	9.4	10.4	[174]
12	Sunitinib	II	Phase II study of Sorafenib Study	Recurrent GBM	32	37.5 mg daily	10.4	9.4	[175]
13	Vandetanib	I/II	Phase I/II Clinical Trial of Vandetanib	Recurrent GBM	32	300 mg daily	6.5	6.3	[176]
14	Bevacizumab (BEV) + TMZ/XRT	III	RTOG 0825 Brain Committee	Newly diagnosed GBM	637	10 mg/kg every 2 weeks + TMZ	Not recorded	15.7	[177]
15	Bevacizumab (BEV) + TMZ/XRT	III	AVAGlio	Newly diagnosed GBM	921	10 mg/kg every 2 weeks + TMZ/XRT	Not recorded	16.9	[140]
16	Bevacizumab (BEV) + TMZ/XRT	III	AVAGlio	Newly diagnosed GBM	463	10 mg/kg every 2 weeks + TMZ/XRT	Not recorded	16.8	[140]
17	Bevacizumab (BEV) + Irinotecan/XRT	II	GLARIUS	Newly diagnosed GBM (MGMT unmethylated)	116	10 mg/kg every 2 weeks + IRI 125 mg/m^2^ every 2 weeks	71.1	16.6	[141]
		II	GLARIUS	Newly diagnosed GBM	54	75 mg/m^2^ TMZ daily/XRT	26.2	17.3	[141]
18	Cilengitide (CIL)	II	The CORE Study	Newly diagnosed GBM/negative MGMT	265	2000 mg twice per weeks + TMZ/XRT	5.6	16.3	[178]
	TMZ/XRT					2000 mg five times per weeks + TMZ/XRT	5.9	14.5	
19	Cilengitide (CIL) + TMZ/XRT	III	The CENTRIC Study	Newly diagnosed GBM/positive MGMT	545	2000 mg twice per weeks + TMZ/XRT	13.5	26.3	[179]
20	Cilengitide (CIL) + TMZ plus Procarbazine/XRT	II	The ExCentric Study	Newly diagnosed GBM/negative MGMT	48	2000 mg twice per weeks + TMZ plus Procarbazine/XRT	30 weeks	58 weeks	[139]

*IRI* irinotecan, *MGMT* O6-methylguanine-DNA methyltransferase, *OS* overall survival, *PFS* progression-free survival, *TMZ* temozolomide, *XRT* radiation therapy]

**Table 3:** The list of clinical trials of VEGF/VEGFR targeting therapeutic targets/agents in GBM patients

Agent/inhibitor	Angiogenic targets	Phase	Tumor type	Combination	Reference
Aflibercept	VEGF-A/B, PIGF	I	rGBM, newly diagnosed GBM, MG	With TMZ and XRT	http://www.clinicaltrials.gov
Bevacizumab	VEGF-A	I/II/III	rGBM	With various combinations	[135, 136, 180]
AEE788	VEGFR1-R2	I/II	rGBM		http://www.clinicaltrials.gov
	EGFR	I/II	rGBM	With Everolimus	http://www.clinicaltrials.gov
Cediranib	VEGFR1 to R3	I/II	Newly diagnosed GBM	With TMZ and XRT	[163]
	PDGFR-β, c-Kit	III	rGBM	Versus Lomustine (randomized trial)	http://www.clinicaltrials.gov
Pazopanib (GW786034)	VEGFR1-R3	II	rGBM		http://www.clinicaltrials.gov
	PDGFR-β, c-Kit	II	rGBM	With Lapanitib	http://www.clinicaltrials.gov
		I	rGBM	With Lapanitib	http://www.clinicaltrials.gov
Sorafenib	VEGFR2-R3	I/II	rGBM, newly diagnosed GBM	With Erlotinib	http://www.clinicaltrials.gov
	BRAF, PDGFR-β, c-Kit, Ras, p38α	I/II	rGBM, newly diagnosed GBM	With Erlotinib, Tipifarnib, or Temsirolimus	http://www.clinicaltrials.gov
		I/II	rGBM	With Temsirolimus	http://www.clinicaltrials.gov
Sunitinib	VEGFR2, PDGFR-β	I	rGBM, rMG	With Irinotecan	http://www.clinicaltrials.gov
	Flt-3, c-Kit	II	rMG		http://www.clinicaltrials.gov
Vandetanib (ZD6474, Zactima®)	VEGFR2, EGFR	I/II	Newly diagnosed GBM	With TMZ and XRT	http://www.clinicaltrials.gov
	RET	I	rMG	With Imatinib and Hydroxyurea	http://www.clinicaltrials.gov
Vatalanib (PTK787)	VEGFR1-R3	I	Newly diagnosed GBM	With TMZ and XRT	[181]
	PDGFR-β and c-Kit	I/II	Newly diagnosed GBM	With TMZ and XRT with or without Vatalanib (randomized trial)	http://www.clinicaltrials.gov
Dastinib	PDGFR-β, Src, BCR-ABL,	I	rMG	With Erlotinib	http://www.clinicaltrials.gov
	c-Kit, EphA2	II	rGBM		http://www.clinicaltrials.gov
Imatinib	PDGFR-β, BCR-ABL, c-Kit	I	rMG	With Everolimus and Hydroxyurea	http://www.clinicaltrials.gov
Tandutinib (MLN518)	PDGFR-β, c-Kit, Flt-3	I/II	rGBM		http://www.clinicaltrials.gov
		II	rMG	With Bevacizumab	http://www.clinicaltrials.gov
Panzem® (2ME2)	HIF-1A	II	rGBM	With TMZ schedule	http://www.clinicaltrials.gov
		II	rGBM		http://www.clinicaltrials.gov
Metronomic TMZ	Endothelial progenitor cells, endothelial cells	II	Newly diagnosed GBM	With gliadel wafer, TMZ, and XRT With Retinoic acid (TMZ with Retinoic acid)	http://www.clinicaltrials.gov
Celecoxib	COX-2	II	GBM with XRT	With Celecoxib, Thalidomide, and Isotretinoin	http://www.clinicaltrials.gov
		II	rMG	Capecitabine, 6-thioguanine with TMZ or Lomustine	http://www.clinicaltrials.gov
Cilengitide	Integrins ανβ3 and ανβ5	I/II	Newly diagnosed GBM	With TMZ and XRT	http://www.clinicaltrials.gov
		III	Newly diagnosed GBM	With TMZ and XRT	http://www.clinicaltrials.gov
		I/II	rMG	With monotherapy (several clinical trials)	http://www.clinicaltrials.gov

*COX-2* cyclo-oxygenase-2, *EGFR* epithelial growth factor receptor, *EIAEDs* enzyme-inducing anti-epileptic drug, *VEGF* vascular endothelial growth factor, *VEGFR* vascular endothelial growth factor receptor, *PDGFR* platelet endothelial growth factor receptor, *HIF1A* hypoxia-inducible growth factor 1 alpha, *PIGF* placental growth factor, *rGBM* recurrent GBM, *rMG* recurrent malignant glioma, *XRT* radiation therapy

The first anti-angiogenesis agent approved for clinical use for brain cancer is a drug called bevacizumab or avastin (Genentech, South San Francisco, CA). Bevacizumab is a monoclonal antibody, and it functions like the physiological antibodies that the human body naturally produces as part of the adaptive immune system. Bevacizumab binds to VEGF and blocks signaling of the molecule and suppresses the formation of new blood vessel growth. Several phase II clinical trials have studied the therapeutic efficacy of bevacizumab as a single agent or in combination with chemotherapy or radiation for recurrent GBM. Bevacizumab as a single agent had significant anti-glioma activity in patients with recurrent glioblastoma [134] (Table 2). A phase I study with a small number of patients suggested that bevacizumab in combination with irinotecan, an inhibitor of topoisomerase I, can be safely administered to patients with malignant gliomas and bevacizumab plus irinotecan achieved a significant improvement in radiographic response (changes in the density of the tumor area) as well as significant increases in progression-free survival among recurrent GBM patients. These studies observed anti-edema induced by bevacizumab treatment augmented the efficacy of the cytotoxic drug by improving the distribution of the drug in these tumors [135, 136]. Previously, the BRAIN study was completed in 2007 for bevacizumab drug trial in recurrent GBM patients, and the outcome of this study was reported the median overall survival (OS) rate of 9.3% and 8.7% with progression-free survival (PFS-6) rate of 43% and 50.3%, respectively, with compared to bevacizumab to bevacizumab plus irinotecan, an inhibitor of topoisomerase I [137]. Later, similar clinical trial was performed by the National Cancer Institute (NCI) for the use of bevacizumab in recurrent GBM patients and they found the median OS rate of 7.8% and PFS-6 rate of 29% [134]. There have been several studies investigated the use of bevacizumab drug target in combination with other drug products to treat recurrent GBM. For example, the BELOB clinical trial was initiated as a randomized phase-II clinical trial and this study used lomustine with bevacizumab or lomustine and bevacizumab alone for the treatment of recurrent GBM patients. The BELOB clinical trial reported that the combination of both drug products (bevacizumab and lomustine) resulted in a PFS-6 of 42% compared to 11% and 18% with OS at 9 months of 12% compared to 7.8% and 8% for lomustine and bevacizumab alone, respectively [138]. Furthermore, based on the BELOB study results, a phase III clinical trial (EORTC 26101) was performed to compare lomustine alone versus lomustine with bevacizumab. In conclusion of the EORTC study, they did find any significant difference in OS for combination treatment versus lomustine alone in recurrent GBM patients [139]. Previously, it has been mentioned that the AVAglio used the revised Response Assessment in Neuro-oncology (RANO) criteria to assess the GBM disease progression in the newly diagnosed GBM patients. The AVAglio clinical trial was performed based on this revised RANO criteria and the clinical trial concluded that bevacizumab prolong the maintenance of performance status in GBM patients, it also reported that decreased in steroid utilization, and prolonged time to deterioration in prespecified cognitive domains of the newly diagnosed GBM patients [140]. Moreover, the similar study has been performed in newly diagnosed GBM patients using the randomized phase II GLAIRUS study, and this study compared the standard care of chemoradiation with temozolomide (TMZ) versus with bevacizumab and irinotecan in GBM patients whose tumors expressed the DNA repair enzyme O6-methyl guanine DNA methyltransferase (MGMT). This phase II GLAIRUS study also concluded that the loss of MGMT increased the sensitivity to therapy with TMZ in newly diagnosed GBM patients [141] (Table 2).

Some of these clinical trials also suggested that the anti-angiogenic therapeutic agents (e.g., VEGF/VEGFR therapeutic targets) (Table 3) enhance the efficacy of conventional treatments by other mechanisms apart from normalization of blood vessels. It was also observed that anti-angiogenic therapy disrupted the tumor vasculature and that the CSC niche microenvironment associated with the tumor blood vessels reduced the CSC which in turn contributes to the efficacy of anti-angiogenic cancer therapy [142]. In another phase II multicenter trial with one hundred sixty-seven patients with recurrent glioblastoma, bevacizumab, alone or in combination with irinotecan, was well tolerated and active in recurrent glioblastoma [137]. It was also suggested that bevacizumab therapy restore a balance between pro- and anti-angiogenic cytokines and induces more stability within the tumor blood vessels with structural and functional phenotype more reflective of normal blood vessels, thus allowing for more effective penetration and distribution of cytotoxic chemotherapeutic drugs within the tumor [143, 144]. A randomized controlled phase II trial of a single-agent bevacizumab or lomustine versus a combination of bevacizumab plus lomustine in patients with recurrent glioblastoma suggested improved OS as compared with monotherapies [138]. Similarly, cediranib (AZD2171, an oral, pan-VEGF receptor inhibitor) as a monotherapy was shown to induce “normalization time window” in tumor vessels in patients with recurrent GBM with significant clinical and functional consequences [145].

## Limitation of Anti-VEGF Therapy and Future Directions

More recent phase 2 trial suggested that the combination of bevacizumab and lomustine did not confer a survival advantage over treatment with lomustine alone in patients with progressive GBM [139]. A randomized phase III trial in newly diagnosed GBM and recurrent grade III gliomas has failed to show an overall survival in [146, 147]. These studies suggest that although a combination of anti-angiogenic therapy with chemotherapy compared with chemotherapy alone produces favorable results with improvements in objective response and PFS in patients with recurrent GBM, a large portion of the patients benefit because of a several factors including changes in the tumor microenvironment (TME) toxicity and resistance. It was proposed that hypoxia caused by vessel regression upregulates hypoxia regulated pro-angiogenic factors like SDF1α leading to recruitment of bone marrow-derived cells (BMDCs) that have the capacity to induce new blood vessel growth leading to tumor progression and relapse [148, 149]. A pro-invasive adaption of the tumors was observed in a subset of GBM patients who had developed multifocal recurrence of tumor’s during anti-VEGF therapy with bevacizumab along with either irinotecan or carboplatin [150, 151]. Toxicity associated with anti-angiogenesis includes thromboembolic and hemorrhagic complications. In addition, gastrointestinal (GI) perforations and one case of reversible posterior leukoencephalopathy were also noted [148, 149]. A compensatory switch to alternative angiogenic pathways could lead to the acquisition of resistance to angiogenic therapy. For example, the PDGF signaling was shown to contribute to angiogenesis in tumors refractory to anti-VEGF treatment by activating tumor stromal cells. However, the anti-tumor effect obtained with a combination of anti-VEGF and anti-PDGF therapy was minimal under conditions of maximal VEGF antagonism, suggesting that inhibition of these two pathways might not be fully additive or synergistic [152].

As discussed above, although anti-VEGF treatment indeed altered several abnormal characteristics of tumor vessels and was generally well tolerated leading to devascularization that limits tumor growth, a large fraction of patients develops toxicity and resistance to this treatment. Moreover, prolonged exposure to anti-angiogenic drugs blocks blood supply to the tumors leading to hypoxic environment which in turn is known to induce chemo-resistance and tumor progression. Therefore, the dose and time of initiation of anti-angiogenic treatment could play a significant role on the therapeutic benefit as the angiogenic inhibitors suppress the tumor growth by inhibiting the growth of blood vessels but does not necessarily kill cancer cells. Single-agent bevacizumab seems to have significant effects on vascular permeability and cerebral edema, suggesting that future trials should focus on the role of bevacizumab as the initial treatment of GBM before starting the chemotherapy treatment [134]. The association between the survival benefit and increased oxygenation leading to vascular normalization in the phase II trials with anti VEGF therapy suggest that identification and validation of early imaging biomarkers and new imaging parameters could help identify the subset of patients who most likely will benefit with anti-angiogenic agents [153]. A baseline of high and low plasma levels of MMP-2 and MMP-9, respectively, were associated with a high response rate and prolonged PFS and OS in recurrent high-grade gliomas treated with Bevacizumab but not with other cytotoxic agents suggesting that it could be predictive biomarker and potentially allow initial patient selection for bevacizumab treatment [154]. Another study showed that the sensitivity to bevacizumab may depend on the relative amount of the various isoforms of VEGF which differ in different molecular weights and biologic properties [155]. Bevacizumab-induced hypertension demonstrated significantly better progression-free survival and OS, suggesting that it could be a physiologic marker of outcome in patients with recurrent GBM [156]. Further, developing patient-specific personalized therapies, based on cellular response of the endothelial cells from the primary brain tumor by screening for sensitivity/resistance to anti-angiogenic agents, can optimize anti-angiogenic therapy in GBM patients [157]. There is also a need to determine novel points of convergence of various signaling pathways in the initiation and development of tumor-induced angiogenesis for predicting and identifying new targets for anti-angiogenic therapy. Several clinical trials are ongoing to validate and expand these efforts, including multiple studies to evaluate non-VEGF anti-angiogenic strategies for malignant glioma patients.
